# Development of the competency scale for primary care managers in Thailand: Scale development

**DOI:** 10.1186/s12875-015-0388-5

**Published:** 2015-12-09

**Authors:** Keerati Kitreerawutiwong, Chanaphol Sriruecha, Wongsa Laohasiriwong

**Affiliations:** Faculty of Public Health Khon Kaen University, 123 Mitraparb Road, Muang District, Khon Kaen, 42000 Thailand; Faculty of Public Health Khon Kaen University, Khon Kaen, Thailand

**Keywords:** Primary care managers, Competency assessment, Validity, Reliability

## Abstract

**Background:**

The complexity of the primary care system requires a competent manager to achieve high-quality healthcare. The existing literature in the field yields little evidence of the tools to assess the competency of primary care administrators. This study aimed to develop and examine the psychometric properties of the competency scale for primary care managers in Thailand.

**Methods:**

The scale was developed using in-depth interviews and focus group discussions among policy makers, managers, practitioners, village health volunteers, and clients. The specific dimensions were extracted from 35 participants. 123 items were generated from the evidence and qualitative data. Content validity was established through the evaluation of seven experts and the original 123 items were reduced to 84 items. The pilot testing was conducted on a simple random sample of 487 primary care managers. Item analysis, reliability testing, and exploratory factor analysis were applied to establish the scale’s reliability and construct validity.

**Results:**

Exploratory factor analysis identified nine dimensions with 48 items using a five-point Likert scale. Each dimension accounted for greater than 58.61 % of the total variance. The scale had strong content validity (Indices = 0.85). Each dimension of Cronbach’s alpha ranged from 0.70 to 0.88.

**Conclusions:**

Based on these analyses, this instrument demonstrated sound psychometric properties and therefore is considered an effective tool for assessment of the primary care manager competencies. The results can be used to improve competency requirements of primary care managers, with implications for health service management workforce development.

**Electronic supplementary material:**

The online version of this article (doi:10.1186/s12875-015-0388-5) contains supplementary material, which is available to authorized users.

## Background

The healthcare system in the 21st century faces challenges because of the complexity inherent in the system. The healthcare managers need to understand the troubling issues in the organization and management of health care delivery [1]. The complexity within the system is an interaction of political, economic, social, demographical changes and the changing patterns of disease [2].

Due to the complexity, Thailand conducted reforms in the healthcare system by implementing universal health coverage policy, reforms on healthcare financing with the process of provider and purchaser split. Thailand also established a community health fund, emphasized primary care services, scaled up district health systems and launched a multidisciplinary team model or the so-called Family Care team [3] to achieve accessibility, equity and quality of healthcare for all citizens.

Healthcare practitioners in the district health system are divided into two levels. At the district level, healthcare is run by health professionals including family physicians, pharmacists, dentists, nurse practitioners, physiotherapy and public health officers who work at community hospitals. At the sub-district level, healthcare staff mainly consists of nurse practitioners, public health officers and public health technical officers who work at Sub-district Health Promoting Hospitals (SHPH) (Previously called Health Centers). SHPHs are located in the sub-district of each province with a total of 9,762 units nationwide [4]. These facilities provided public health services and primary care to people registered under the universal health coverage scheme. The manager of the primary care facility is called Sub-district Health Promoting Hospital Director (SHPH-D), who acts as a primary care manager in his or her facility.

The SHPH-Ds are front line managers, responsible for planning, organizing, staff recruiting, directing, controlling and coordinating with all organizations in a district [5] in line with implementing policy into practice in primary care services through the communication with health practitioners and all stakeholders. SHPH-Ds have been given an important responsibility of ensuring quality health care provided to the rural population in Thailand. Therefore, they need to have an appropriate level of competency to achieve the goal.

The definition of competency includes motivation, personal traits, skills, and aspects of one’s self image or social roles, knowledge that underlies leadership and management actions [6–9]. In the literature review instruments of assessment of healthcare executives by the American College of Healthcare Executives [10] and The Healthcare Leadership Alliance (HLA) [11] are documented as well as a study on competency among middle and senior managers in community health services in Australia [12]. The existing instruments provide an assessment of a manger’s competency in developed countries based on different healthcare systems which are not appropriate for adaptation in the Thai context.

Moreover, there exists a literature of competency among Master of Public Health graduate students [13], healthcare personnel who practice at the primary care level such as primary care providers and nursing staff in community health care [14, 15]. In addition, the Office of the Civil Services Commission’s competency provided a framework for assessing administrative positions [16]; however, this framework is not appropriate for SHPH-Ds because its assessment tool is for the administrator in all disciplines, not specific to the job/role of the managers in primary care. In line with the study of Chouh-Jiaun Lin (2010), it was recommended that similar questionnaires should not be used to measure public health nurses and head nurses [17]. Hence, the studies conducted on the primary care manager competency at sub-district level in Thailand were still limited.

WHO (2007) recommended that all countries build leadership and management capacity in health on the four dimensions as follows: 1) Ensuring adequate numbers of managers, 2) Ensuring that managers possess an appropriate degree of competency, 3) Creating better critical management support systems and 4) Creating an enabling working environment [18]. Competency framework in the context of Thailand and a psychometric properties tool are important for developing job competency and improving the performance of SHPH-Ds. This study aimed to fill this gap in the existing knowledge of the psychometric properties tool for assessing primary care manager competencies in Thailand. This paper describes the development and testing of a new survey instrument using a qualitative approach reinforced by quantitative methodology.

## Methods

### Design

The instrument development model combining qualitative and quantitative approaches was used as a methodological study design. The study consisted of two phases: (1) development of questionnaire and (2) evaluation of its psychometric properties.

### Ethical considerations

This study has been granted ethical approval by the Khon Kean University Ethics committee (Code no. HE562069). All participants were informed about the purposes and the methods of the study beforehand. The protection of human subjects was assured by the use of two consent forms in both phases. The participants who agreed to participate in the study were asked to sign a written consent form document. The anonymity of the participants was ensured. Moreover, prior to data collection, the permission for research from the Provincial Health Office and District Health Office in five provinces of Phitsanulok, Utaradit, Sukothai, Petchaboon, and Tak (Health Region 2) were granted. The two stages of the study procedure are described below.

### Study Framework

#### Phase 1: Development of questionnaire

##### Step 1

We used the systematically developed definition of competency of primary care managers at the sub-district level in Thailand. The dimensions of competency criteria were determined by in-depth interviews and focus group discussions. Purposive sampling was used in the recruitment of 35 stakeholders to cover all perspectives in order to ensure the recording of all of the voices of key informants. The criteria were based on their expertise in primary care for at least one year and familiarity with the primary care services. All stakeholders were selected from policy makers, academics, practitioners, lay health workers, and consumer representatives. The data were collected from all stakeholders, including six policy makers, five academicians, two chief executive of the sub-district administrative organization, three primary care providers in sub-district health promoting hospitals, five sub-district health promoting hospital directors, eight patients and nine village health volunteers.

The in-depth interviews were used to extract information from the health professionals and local organizations to allow them to express their deepest thoughts about a certain subject, while the focus group discussions were used to extract information from less opinionated groups, lay health workers and patients, since this technique allows them to listen to each other’s ideas and build upon them to generate the dynamics of this group. The semi-structure guideline was used in collecting data. Each interview was audio-recorded, and the interviewer took notes during the interview. In-depth interviews were performed ranging in duration from 47 to 64 min and focus group discussions lasted from 72 to 88 min. Both the interviews and focus group discussions were conducted until no new issue emerged. All the sessions were transcribed verbatim for analysis. Trustworthiness was achieved through use of credibility, triangulation, and member checking and peer debriefing. The results of content analysis revealed the nine dimensions were as follows: 1) leadership, 2) communication, 3) partnership, 4) system thinking and strategic decision making, 5) organizational development and professionalism, 6) emotional intelligence, 7) proactive approach, 8) financial planning and 9) information management.

##### Step 2

An item pool was generated from the conceptual structure of nine competencies from step 1, yielding 123 items in health manager competency [5, 12]. Likert scale was designed to assess perceived degree of individual competency. It is appropriate for the construction of clear terms, and practical for self-administrated surveys. A 5-point Likert scale, ranging from 1 (“novice” = SHPH-Ds having little or no knowledge/ability, or no previous experience of the competency described) to 5 (“expert” = SHPH-Ds are the primary sources of knowledge and information in the management in primary care), with neutral in the middle, which was selected based on consideration of reliability of measurement that a rating scale with fewer than five scales points should be avoided [19] with consideration given to a balanced number of positive and negative response options [20]. Each item in the questionnaire was assessed by the 5-point Likert scale, which has the ability to discriminate between the response levels and reduce the burden of tasks performed by respondents when compared to higher-level scales [21]. Scores for each dimension were combined and transformed to a 100 point total in order to make the results practical to compare and interpret across dimensions. A higher score indicates a better competency.

Next, the content validity tests were performed by seven experts not included in the 35 stakeholders in step 1, who were asked (1) to give suggestions on the relevancy and overlapping of each item to the definition, (2) to evaluate clarity and conciseness of the wordings, and (3) to point out any missing items that should be included. These experts with diverse skills and expertise were asked to evaluate the content validity of the instrument, purposively recruited from the area of interest of this study. Among them were two policy makers with expertise in human resources management, two lecturers with expertise in human workforce development in primary care and community health, two practitioners and an expert in measurement development. A value of item-level content validity index (I-CVI) was conducted. The evaluation followed the process suggested by Polit, Beck, and Owen (2007) in having experts rate each item on a 4-point Likert scale (not relevant, somewhat relevant, quite relevant, and very relevant) based on item clarity and conciseness. Raters were asked to provide comments and suggestions for revising or adding new items. The ratings were used to calculate an item-level content validity index (I-CVI) and to determine if items should be revised or deleted [22]. A criterion of 0.80 of I-CVI among the experts were selected for inclusion in the list of items [22]. Totally, 39 items were deleted regarding an acceptable value and 84 items were retained.

##### Step 3

A pilot test of the preliminary instrument with a 5-point Likert scale was conducted on primary care managers in the other regions that have similar characteristics of sample in Phichit province (Region health 3). The clarity and difficulty of the items as well as suggestions of the instrument were included, which was sent to 41 samples to complete the pilot survey. Cronbach’s alpha coefficient was examined to determine the internal consistency of the scale which indicates how well the items fit together conceptually [23, 24], with the acceptable value of ≥0.70 [21]. If an instrument contains two or more subscales, Cronbach’s alpha should be computed for each subscale as well as the entire scale [23, 24]. Therefore, Cronbach’s alpha was computed for both overall and each subscale. In addition, internal consistency was also assessed by corrected item-total correlation, indicating the magnitude of association for individual items with the total scale [21, 25]. The corrected item-total had acceptability with the value above 0.2 [21]. The Cronbach’s alpha coefficient ranged from 0.74 to 0.82, indicating an achieved minimum reliability of 0.70 for the new instrument [25]. The item-total correlation ranged from 0.20 to 0.74, indicating no item redundancy. All 84 items were retained because met all criteria for the acceptance of internal consistency and corrected item-total correlation.

#### Phase 2: Evaluation of its psychometric properties

A field-test was conducted for evaluation of the reliability and validity of the instrument. In Step 4, the field test for psychometric properties of instrument was conducted in a large sample of primary care managers. Moreover, exploratory factor analysis was performed to evaluate the construct validity.

### Sample

A sample of 620 SHPH-Ds working at the sub-district level in five provinces was selected by using a simple random sampling method. The questionnaires were distributed to the 525 sampled, 509 were returned with a 96.95 % return rate. Among the 509 returned questionnaires, 18 (3.54 %) were incomplete, and 4 (0.79 %) gave the same score for all 84 items. Finally, a total of 487 questionnaires were analyzed.

### Data analysis

Statistical analysis was carried out using SPSS 20.0 software. Internal consistency and item analysis were assessed by calculating the Cronbach’s a coefficient. An exploratory factor analysis using principal component method with Promax rotation was used to explore the structure of the items and examine its construct validity because it allows correlation of the factors [26]. Four criteria used in retaining items and in the determination of the factors were that (1) a factor loading should be greater than 0.30 [26, 27] in order to maximize differentiation between factors, (2) there should be no or few item cross loadings by examining both the highest and second highest factor loadings, with a difference of less than 0.2 between the two loadings in order to show sufficiently large correlation in each factor [28], (3) each retained factor should have at least three items and (4) all retained items should share the same conceptual meaning [26, 29, 30]. Finally, the final draft of the primary care manager competency scale was deleted 36 items, and there remained 48 items within nine dimensions.

## Results

### Demographic characteristics of samples

Among the 487 samples, there were adults aged 24 to 60 years (mean = 47.34, SD = 7.12) with 58.11 % being female. 70.02 % graduated with Bachelor Degrees of Public Health Sciences. The length of working experience of those in charge of sub-district health promoting hospital directors ranged from 1 to 32 years with an average of 13.21 (SD = 9.18) years (Table 1).

**Table 1:** Personal Characteristics (*n* = 487)

Personal Characteristics	Number	Percent
Gender		
Male	283	58.11
Female	204	41.89
Marital Status		
Single	45	9.24
Married	384	78.85
Widowed/Divorced/Separated	58	11.91
Age (years)		
<35	24	4.93
36–40	71	14.58
41–45	92	18.89
46–50	108	22.18
51–55	142	29.16
56–59	50	10.27
(Mean = 47.36, SD = 7.14)		
Highest Educational level		
Certificate	60	12.32
Bachelor degree	341	70.02
Master degree	86	17.66
Educational background		
Public Health	381	78.23
Nurse	82	16.84
Others (Other health sciences such as public health officers, dental hygienist, pharmacy assistant etc.)	24	4.93
Working experience in charge of sub-district health promoting hospital directors (years)		
<5	101	20.74
6–10	138	28.34
11–15	80	16.43
16–20	70	14.37
21–25	35	7.19
26–30	34	6.98
>30	29	5.95
(Mean = 13.21, SD = 9.18)		
Experience in training		
No	110	22.59
Yes^a^	377	77.41
- Sub-district Health Promotion Hospital Director Program	284	75.33
- First-line Public Health Administrators Training Program	229	60.74
- Other curriculums (such as Middle Level Public Health Administrators Training Program, Modern Manager Training course)	48	12.73

^a^more than 1 answer is acceptable

### Construct validity

Within nine dimensions, the results of the exploratory factor analysis with promax rotation showed a scale comprised of 48 total items. Barlett’s test of sphericity was significant (*x*^2^ = 11,243.939, *p* = 0.000), and KMO value was 0.958. The results of factor analysis are showed in Table 2. Finally, nine dimensions were retrieved, which together accounted for 58.61 % variance, explained with eigenvalue from 1.01 to 16.38. The factor loadings were from 0.30 to 0.93. The overall scale Cronbach’s alpha was 0.96 with dimensions Cronbach’s alpha from 0.70 to 0.88. The final version of questionnaire is shown in Additional file 1.

**Table 2:** Factor loading and item statements (*n* = 487)

Dimensions and Item statements	Factor Loading
Dimension 1 Leadership	
(6 items with Eigenvalue = 1.69, % of Variance = 3.51, Cronbach’s Alpha Coefficient = 0.84)	
i1 Clarify vision, mission, and goal of SHPH precisely	0.86
i3 Understand policy and communicate the policy to staff to implement it into practice.	0.73
i2 Innovate and create works to achieve performance of SHPH	0.70
i4 Integrate mission of SHPH to community	0.52
i7 Give advice and coaching to health workers in detailed steps.	0.45
i9 Conduct performance assessment on the health workers on the basis of good governance.	0.43
Dimension 2 Communication	
(5 items with Eigenvalue = 1.01, % of Variance = 2.10, Cronbach’s Alpha Coefficient = 0.70)	
i13 Provide clear information, news and knowledge on healthcare to patients and the public.	0.72
i15 Possess effective communication skills to convince the public and the community to cooperate with the SHPH in order to achieve the goals.	0.63
i14 Provide accurate information on public health problems to policy makers.	0.40
i17 Report the performance of the SHPH to the public and private sectors and the community according to the KPI.	0.33
i16 Maintaining mutual understanding and trust with clients, communities and other team members through effective communication.	0.30
Dimension 3 Partnership	
(4 items with Eigenvalue = 1.38, % of Variance = 2.87, Cronbach’s Alpha Coefficient = 0.75)	
i21 Persuade and cooperate with other organizations to support innovative health projects or activities in the community.	0.58
i25 Search for support, resources and staff exchanges from other organizations and effectively employ them in health service work in the area.	0.54
i26 Work effectively through intersectional collaboration with community hospitals, local government organizations, and community groups and parties.	0.54
i28 Encourage the community to participate in solving health problems and monitor public health risk.	0.40
Dimension 4 System thinking and strategic decision making	
(9 items with Eigenvalue = 16.38, % of Variance = 34.13, Cronbach’s Alpha Coefficient = 0.86)	
i39 Use evaluation feedbacks to improve the quality of working staff.	0.70
i36 Design appropriate strategies to improve the quality of SHPH.	0.69
i31 Apply information on health care and other related ones appropriately to identify public health problems.	0.61
i34 Apply appropriate techniques to solve community health problem.	0.56
i37 Implement plans and projects appropriately according to the nature of public health problems.	0.56
I38 Monitor staff’s performance efficiency according to the KPI.	0.54
i35 Able to predict and plan man power needs that respond to the health promotional plan of the SHPH.	0.48
I32 Able to analyze factors or situations affecting public health problems.	0.46
I33 Provide guidelines, working procedure, projects and work activities that match the policy of SHPH.	0.41
Dimension 5 Organizational development and professionalism	
(6 items with Eigenvalue = 1.96, % of Variance = 4.09, Cronbach’s Alpha Coefficient = 0.86)	
i48 Be courteous with the seniors both in SHPH and community.	0.91
i49 Maintain confidentiality of clients.	0.84
i45 Focus on the benefits of clients and the public.	0.72
i47 Respect the judgment of the working staff and other local organizations.	0.65
i46 Give advice on ethical practices and related laws on how to protect clients’ rights.	0.56
i43 Work hard, be responsible and achieve reliable and satisfactory performance acknowledged by others.	0.31
Dimension 6 Emotional intelligence	
(3 items with Eigenvalue = 1.10, % of Variance = 2.30, Cronbach’s Alpha Coefficient = 0.78)	
i54 Listen to others ideas, suggestions and opinions.	0.80
i53 Control and manage emotions appropriately.	0.65
i51 Accept the results of performance evaluation.	0.51
Dimension 7 Proactive approach	
(4 items with Eigenvalue = 1.32, % of Variance = 2.76, Cronbach’s Alpha Coefficient = 0.88)	
i61 Promote healthy lifestyle to all groups of people in the community.	0.92
i62 Encourage the process of building healthy public policies in community.	0.81
i60 Empower clients to be able to respond to their health problems.	0.77
i63 Establish processes to promote a strong community with an awareness of health as a public concern.	0.43
Dimension 8 Financial planning	
(3 items with Eigenvalue = 1.13, % of Variance = 2.35, Cronbach’s Alpha Coefficient = 0.82)	
i74 Provide cost-effective public health programs	0.93
i69 Use the mechanism of The SHPH administrative committee to gain financial support.	0.89
i72 Manage financial risks effectively.	0.36
Dimension 9 Information management	
(8 items with Eigenvalue = 2.17, % of Variance = 4.52, Cronbach’s Alpha Coefficient = 0.83)	
i79 Manage information system to achieve fast and convenient access.	0.70
i82 Share information between providers and network to improve healthcare.	0.66
i78 Apply information technology to serve health needs of clients in the community.	0.63
i81 Advice for public health practitioner on data analysis	0.63
i80 Collect data and information on health problems systematically to be analyzed.	0.59
i83 Communicate news and information on health issues through community media or networks.	0.57
i84 Search information on public health or on epidemiology to be used in health and project planning.	0.52
i75 Design and plan an accurate and reliable community health information management.	0.39

### Reliability of the final version

The results of internal consistency analysis for each dimension are presented in Table 3. The overall scale internal consistency of Cronbach’s alpha was 0.96, and for each dimension ranged from 0.70 to 0.88.

**Table 3:** Internal consistency of the competency scale for sub-district health promoting hospital directors as estimated by Cronbach’s alpha

Dimensions	Number of items	Range of item-total correlations	Cronbach’s alpha	Range of alphas if individual item deleted
1. Leadership	6	0.54–0.68	0.84	0.80–0.83
2. Communication	5	0.22–0.58	0.70	0.60–0.77
3. Partnership	4	0.52–0.57	0.75	0.67–0.70
4. System thinking and strategic decision making	9	0.52–0.66	0.86	0.84–0.85
5. Organizational development and professionalism	6	0.38–0.75	0.86	0.83–0.89
6. Emotional intelligence	3	0.57–0.65	0.78	0.66–074
7. Proactive approach	4	0.61–0.80	0.88	0.82–0.89
8. Financial planning	3	0.54–0.76	0.82	0.66–0.89
9. Information management	8	0.49–0.61	0.83	0.80–0.82
Total	48		0.96	

## Discussion

This current study examined the factor structure and psychometric properties in a large sample of primary care managers and the results strongly suggested that the instrument was a validated self-report scale for assessing competency of primary care managers. The description of the dimension and definition of competency was systematically developed for the specific context and translated in measurable terms to create an instrument similar to those in previous studies [5, 31]. In consonance with the suggestion of Johnson & Onwuegbuzie . J. (2010), mixed research techniques provide a rigorous way for developing quantitative instruments [32].

Content validity illustrates the scale’s quality because it ensures congruence between dimension content and data collection tool [33]. Results of item validation showed the value of CVI is 0.85, with accepted standards of CVI of 0.80 or greater [33–35].

The results of Cronbach’s alpha and item-total correlation show a good homogeneity. The ranging of each dimension of Cronbach’s alpha was 0.70 to 0.88. It can be concluded that the items in each dimension are adequate samples of content represented in each dimension [23]. Item-total correlation ranged from 0.22 to 0.67, which was considered acceptable. It demonstrated that items in each dimension were in correlation with each other [36] and did not show redundancy [25].

Exploratory factor analysis indicating nine dimensions were extracted with 58.61 % of the total percentage of variance explained, which was acknowledged as adequate in capturing the main features of the phenomenon [29]. The number of items in each dimension ranged from 3 to 10 items and was in line with the study of Hair et al. (2013) which indicated that each dimension should have at least 3 items [27].

The finalized instrument is similar to the other studies in terms of leadership, communication, partnership, system thinking and strategic decision making, proactive approach, and information management [5, 11]. Since the SHPH-Ds are primary health care managers in the health care service system of the country, their responsibility is to implement the regional and national policy into practices in their community. They communicate with and supervise their subordinates, cooperate and coordinate with other organizations in the area, monitor work plans and financial plans and give advice to subordinates [37]. The SHPH-Ds performed their work under resource-constraints. They needed a systematic and proactive managing planning, for example, disease prevention, health promotion; and they needed empirical data in the community in order to control budgets. Moreover, this study illustrates the difference in competency between the competency of Master of Public Health Program [13] and competency of primary care managers that support the evidence on differentiation of competency in different positions. SHPH-Ds deliver public health and primary care in Thailand, they act as managers and practitioners in the complex situation therefore, a set of competency on organizational development and professionalism, emotional intelligence, proactive approach, financial planning and information management were found in the real practice. Therefore, measurement of competency in different job positions requires a different instrument with the ability to be applicable as a means to improve competency for specific job positions.

In addition, the dimension of organizational development and professionalism and financial planning were consistent with The Healthcare Leadership Alliance and the American College of Healthcare Executives [11]. At present, health care service system is client-centered. For clients to receive quality service, health care providers need to maintain professional and ethical standards, and keep up with the most up-to date knowledge and information. The objective is control of quality and standards in the performance of public health officers nationwide [38].

As for the dimension of emotional intelligence derived from this study, owing to the fact that in the Thai healthcare system, scarcity of resources is a common phenomenon, primary care managers need to cooperate with others to build networks for a successful operation. The managers therefore need to have the sensitivity to recognize these human issues and to act on them in an effective manner, requires a leader with high emotional intelligence when they interact with the others [39]. Effective collaboration requires both technical expertise and emotional intelligence. Moreover, Judith Daire, Lucy Gilson and Susan Cleary (2014) described of three types of intelligence for healthcare managers including cognitive intelligence, emotional intelligence and social intelligence [40]. These findings are consistent with the study of Smith K.B., Profetto-McGrath G. & Cummings G. G. (2009) which recommended that emotionally intelligent leaders influence employee retention, quality of patient care and patient outcomes [41]. Therefore, emotional intelligence evidence requires development in education and training in healthcare services. In Thailand, emotional intelligence is one element of competency in the District Health System Management Learning Project (DHML) [42]. This competency facilitates health managers’ work in a complex system, and in order to maintain job happiness, they need to possess self-awareness and a balance between work and family life [43].

## Conclusions

The instrument was systematically developed with 9-dimensions, comprised of 48-items to assess competency of primary care managers in Thailand. The psychometric properties show that this instrument is a validated tool. The results have several implications for primary care managers, policy makers and educators. For primary care managers, they can use the instrument to assess their competency for feedback information which might lead to a higher level of awareness of their competency level and career opportunity. The usefulness for policy makers are in the areas of performance appraisals, recruitment, identification of training needs for continuing education and formulation of a competency based curriculum or program in human resource management. Moreover, this competency model is recommended to be integrated into the planning of frontline managers at higher level administration in primary care in Thailand.

## Limitations

The limitations of this study are as follows: Firstly, some key informants (clients) were selected in region health 2, whereas the other key informants from other sectors, for example, policy makers and academicians, were chosen from the other regions and national levels to fulfill the competency framework. The qualitative findings indicated that the perceptions of the key informants in each level contained differences and they fulfill qualitative data. Secondly, the instrument in this study is specified for primary health care service in Thailand which corresponds to the Thai context. Therefore, the instrument must be tested and adjusted before implementation in other contexts. Despite the aforementioned shortcomings, this instrument is a valuable tool for capturing the competency of primary care manager in Thailand and serves as a starting point in how to measure SHPH-Ds competency. The repetitive use of the instrument in other regions in order to validate the instrument is needed.

For further study, a cross-sectional study is recommended to explore the relationship between SHPH-Ds competency toward quality of care and health outcomes. In addition, a study on factors affecting the SHPH-Ds’ competencies, designing competency based training programs, assessment of the competency based training programs for the SHPH-Ds and the retention of the competencies in the SHPH-Ds are suggested for further studies to provide more evidence for primary care managers.

## Additional file

Additional file 1:
**The final version of primary care manager competency assessment questionnaire.** (DOC 147 kb)

## Abbreviations

SHPH: Sub-district Health Promoting Hospital
SHPH-D: Sub-district Health Promoting Hospital Director
I-CVI: Item-level content validity index
CVI: Content validity index
