# Supplementary material for: Development of the competency scale for primary care managers in Thailand: Scale development
Source: BMC Fam Pract. 2015 Dec 9;16:174. doi: 10.1186/s12875-015-0388-5 (PMC4673780; doi:10.1186/s12875-015-0388-5)
Supplement: Additional file 1: — The final version of primary care manager competency assessment questionnaire. (DOC 147 kb) [file 12875_2015_388_MOESM1_ESM.doc]

**Primary care manager competency assessment questionnaire**

**Instructions**: This questionnaire is used for assessing the competency of primary care manager; it consists of two sections: section 1, personal characteristics and section 2, the competency of primary care manager.

**Section 1: Personal Characteristics**

*Instruction:* Please mark “ ✓ ” in “ ❑ ” for best answer or write your answer on the line provided.

1. Gender ❑ Male ❑ Female

2. Marital status ❑ Single ❑ Married ❑ Widowed

❑ Divorced ❑ Separated

3. Age ____________ years

4. Highest Educational level

❑ Certificate

❑ Bachelor degree

❑ Master degree

❑ Doctoral degree

5. Educational background

❑ Public Health

❑ Nurse

❑ Other (identify) ___________________________

6. Working experience in charge of sub-district health promoting hospital directors _______ years

7. Experience in training

❑ No

❑ Yes (multiple responses)

❑ Sub-district Health Promotion Hospital Director Program

❑ First-line Public Health Administrators Training Program

❑ Middle Level Public Health Administrators Training Program

❑ Other (identify) ____________________________________

**Section 2: The competency of primary care manager.**

*Instruction:* Please mark “ ✓ ” in “ ❑ ” for best answer on the line competency. The scale is classified into 5 levels: 1 = Novice knowledge/skills, 2 = Approaching proficiency, 3 = Fully proficient,

4 = Approaching advanced, and 5 = Advanced/expert.

| **Dimensions and Item statements** | **Competency scale** | | | | |
| --- | --- | --- | --- | --- | --- |
|  | **Novice knowledge/skills** | **Approaching proficiency** | **Fully proficient** | **Approaching advanced** | **Approaching advanced** |
|  | **[1]** | **[2]** | **[3]** | **[4]** | **[5]** |
| **Dimension 1 Leadership** | | | | | |
| 1. Clarify vision, mission, and goal of SHPH precisely | ❑ | ❑ | ❑ | ❑ | ❑ |
| 2. Understand policy and communicate the policy to staff to implement it into practice. | ❑ | ❑ | ❑ | ❑ | ❑ |
| 3. Innovate and create works to achieve performance of SHPH | ❑ | ❑ | ❑ | ❑ | ❑ |
| 4. Integrate mission of SHPH to community | ❑ | ❑ | ❑ | ❑ | ❑ |
| 5. Give advice and coaching to health workers in detailed steps. | ❑ | ❑ | ❑ | ❑ | ❑ |
| 6. Conduct performance assessment on the health workers on the basis of good governance. | ❑ | ❑ | ❑ | ❑ | ❑ |
| **Dimension 2 Communication** |  |  |  |  |  |
| 7. Provide clear information, news and knowledge on healthcare to patients and the public. | ❑ | ❑ | ❑ | ❑ | ❑ |
| 8. Possess effective communication skills to convince the public and the community to cooperate with the SHPH in order to achieve the goals. | ❑ | ❑ | ❑ | ❑ | ❑ |
| 9. Provide accurate information on public health problems to policy makers. | ❑ | ❑ | ❑ | ❑ | ❑ |
| 10. Report the performance of the SHPH to the public and private sectors and the community according to the KPI. | ❑ | ❑ | ❑ | ❑ | ❑ |
| 11. Maintaining mutual understanding and trust with clients, communities and other team members through effective communication. | ❑ | ❑ | ❑ | ❑ | ❑ |
| **Dimensions and Item statements** | **Competency scale** | | | | |
|  | **Novice knowledge/skills** | **Approaching proficiency** | **Fully proficient** | **Approaching advanced** | **Approaching advanced** |
|  | **[1]** | **[2]** | **[3]** | **[4]** | **[5]** |
| **Dimension 3 Partnership** |  |  |  |  |  |
| 12. Persuade and cooperate with other organizations to support innovative health projects or activities in the community. | ❑ | ❑ | ❑ | ❑ | ❑ |
| 13. Search for support, resources and staff exchanges from other organizations and effectively employ them in health service work in the area. | ❑ | ❑ | ❑ | ❑ | ❑ |
| 14. Work effectively through intersectional collaboration with community hospitals, local government organizations, and community groups and parties. | ❑ | ❑ | ❑ | ❑ | ❑ |
| 15. Encourage the community to participate in solving health problems and monitor public health risk. | ❑ | ❑ | ❑ | ❑ | ❑ |
| **Dimension 4 System thinking and strategic decision making** | | | | | |
| 16. Use evaluation feedbacks to improve the quality of working staff. | ❑ | ❑ | ❑ | ❑ | ❑ |
| 17. Design appropriate strategies to improve the quality of SHPH. | ❑ | ❑ | ❑ | ❑ | ❑ |
| 18. Apply information on health care and other related ones appropriately to identify public health problems. | ❑ | ❑ | ❑ | ❑ | ❑ |
| 19. Apply appropriate techniques to solve community health problem. | ❑ | ❑ | ❑ | ❑ | ❑ |
| 20. Implement plans and projects appropriately according to the nature of public health problems. | ❑ | ❑ | ❑ | ❑ | ❑ |
| 21. Monitor staff’s performance efficiency according to the KPI. | ❑ | ❑ | ❑ | ❑ | ❑ |
| 22. Able to predict and plan man power needs that respond to the health promotional plan of the SHPH. | ❑ | ❑ | ❑ | ❑ | ❑ |
| 23. Able to analyze factors or situations affecting public health problems. | ❑ | ❑ | ❑ | ❑ | ❑ |

| **Dimensions and Item statements** | **Competency scale** | | | | |
| --- | --- | --- | --- | --- | --- |
|  | **Novice knowledge/skills** | **Approaching proficiency** | **Fully proficient** | **Approaching advanced** | **Approaching advanced** |
|  | **[1]** | **[2]** | **[3]** | **[4]** | **[5]** |
| 24. Provide guidelines, working procedure, projects and work activities that match the policy of SHPH. | ❑ | ❑ | ❑ | ❑ | ❑ |
| **Dimension 5 Organizational development and professionalism** | | | | | |
| 25. Be courteous with the seniors both in SHPH and community. | ❑ | ❑ | ❑ | ❑ | ❑ |
| 26. Maintain confidentiality of clients. | ❑ | ❑ | ❑ | ❑ | ❑ |
| 27. Focus on the benefits of clients and the public. | ❑ | ❑ | ❑ | ❑ | ❑ |
| 28. Respect the judgment of the working staff and other local organizations. | ❑ | ❑ | ❑ | ❑ | ❑ |
| 29. Give advice on ethical practices and related laws on how to protect clients’ rights. | ❑ | ❑ | ❑ | ❑ | ❑ |
| 30. Work hard, be responsible and achieve reliable and satisfactory performance acknowledged by others. | ❑ | ❑ | ❑ | ❑ | ❑ |
| **Dimension 6 Emotional intelligence** | | | | | |
| 31. Listen to others ideas, suggestions and opinions. | ❑ | ❑ | ❑ | ❑ | ❑ |
| 32. Control and manage emotions appropriately. | ❑ | ❑ | ❑ | ❑ | ❑ |
| 33. Accept the results of performance evaluation. | ❑ | ❑ | ❑ | ❑ | ❑ |
| **Dimension 7 Proactive approach** | | | | | |
| 34. Promote healthy lifestyle to all groups of people in the community. | ❑ | ❑ | ❑ | ❑ | ❑ |
| 35. Encourage the process of building healthy public policies in community. | ❑ | ❑ | ❑ | ❑ | ❑ |
| 36. Empower clients to be able to respond to their health problems. | ❑ | ❑ | ❑ | ❑ | ❑ |
| 37. Establish processes to promote a strong community with an awareness of health as a public concern. | ❑ | ❑ | ❑ | ❑ | ❑ |

| **Dimensions and Item statements** | **Competency scale** | | | | |
| --- | --- | --- | --- | --- | --- |
|  | **Novice knowledge/skills** | **Approaching proficiency** | **Fully proficient** | **Approaching advanced** | **Approaching advanced** |
|  | **[1]** | **[2]** | **[3]** | **[4]** | **[5]** |
| **Dimension 8 Financial planning** | | | | | |
| 38. Provide cost-effective public health programs | ❑ | ❑ | ❑ | ❑ | ❑ |
| 39. Use the mechanism of The SHPH administrative committee to gain financial support. | ❑ | ❑ | ❑ | ❑ | ❑ |
| 40. Manage financial risks effectively. | ❑ | ❑ | ❑ | ❑ | ❑ |
| **Dimension 9 Information management** | | | | | |
| 41. Manage information system to achieve fast and convenient access. | ❑ | ❑ | ❑ | ❑ | ❑ |
| 42. Share information between providers and network to improve healthcare. | ❑ | ❑ | ❑ | ❑ | ❑ |
| 43. Apply information technology to serve health needs of clients in the community. | ❑ | ❑ | ❑ | ❑ | ❑ |
| 44. Advice for public health practitioner on data analysis | ❑ | ❑ | ❑ | ❑ | ❑ |
| 45. Collect data and information on health problems systematically to be analyzed. | ❑ | ❑ | ❑ | ❑ | ❑ |
| 46. Communicate news and information on health issues through community media or networks. | ❑ | ❑ | ❑ | ❑ | ❑ |
| 47. Search information on public health or on epidemiology to be used in health and project planning. | ❑ | ❑ | ❑ | ❑ | ❑ |
| 48. Design and plan an accurate and reliable community health information management. | ❑ | ❑ | ❑ | ❑ | ❑ |

**Thank you for your kind cooperation in completing this questionnaire**
